# Supplementary material for: Reconstructing smoking history through dental cementum analysis - a preliminary investigation on modern and archaeological teeth
Source: PLoS One. 2025 May 27;20(5):e0323812. doi: 10.1371/journal.pone.0323812 (PMC12111438; doi:10.1371/journal.pone.0323812)
Supplement: Table S2 — Summary of the chi-squared and p-values between cementum width and smoking habits and damage, in overall cohort, modern and archaeological subsamples. (DOCX) [file pone.0323812.s002.docx]

| **Kruskal-Wallis rank sum test** | | |
| --- | --- | --- |
| **Modern Samples** | **Chi-squared** | **p-value** |
| Width ~ Smoking Habits | 5.99 | 0.05 |
| Width ~ Smoking Damage | 8.65 | 0.01 |
| **Archaeological Samples** | **Chi-squared** | **p-value** |
| Width ~ Smoking Habits | 6.78 | 0.08 |
| Width ~ Smoking Damage | 7.78 | 0.02 |
| **Overall Cohort** | **Chi-squared** | **p-value** |
| Width ~ Smoking Habits | 10.14 | 0.04 |
| Width ~ Smoking Damage | 11.71 | 0.002 |
