# Supplementary material for: Reconstructing smoking history through dental cementum analysis - a preliminary investigation on modern and archaeological teeth
Source: PLoS One. 2025 May 27;20(5):e0323812. doi: 10.1371/journal.pone.0323812 (PMC12111438; doi:10.1371/journal.pone.0323812)
Supplement: Attachment — Submitted filename: Feedback.docx [file pone.0323812.s003.docx]

The paper represents a novel approach to the study of smoking status in past populations with the use of cementochronology. The authors provided an in-depth literature review on the topic including the answers that AEFC can provide to specific osteoarchaeological questions. The study provided a preliminary investigation using a sound methodology that has the potential to be applied to other future studies. The Discussion provided a good interpretation of the results. It also addressed the issues found with the archaeological sample and suggested areas of future potential development. Overall, the paper represents a great contribution to the field although some aspects of the Materials-Methods and Results could be explained and/or presented better.

-The **Materials and Methods** defined the archaeological samples in Lines 121-122 (Smokers, Potential smokers, Indet, Non-smoker) but the modern samples were defined in the **Results** (smokers, non-smokers and ex-smokers). I have to admit that I had to read these sections more than once to understand what sample was being referred to and how. This is the reason I believe the Materials and Methods – and Results would be better presented had these terms been explained in one place (the methods). If the donors' self-reported status is listed/defined in the methods, then the results section could present the data according to the cementochronology.

-Also, the term ‘smoking damage’ is defined in Line 238 but sometimes ‘damage’ was used interchangeably to mean ‘smoking damage’. I’d recommend sticking to this term instead of just calling it ‘damage’ because the word ‘damage’ was used in other sections of the paper to mean different things. So, to not create confusion I’d recommend you to state the full word ‘smoking damage’ to make a clear distinction from other meanings.

**Discussion:**

-The discussion mentions that recent studies in South Korea and China highlighted the relationship between air pollutants and the development of periodontitis. The assumption that your archaeological samples belonged to individuals who lived during the Industrial Revolution implied that they would have been exposed to environmental pollution. Now, specifically to your archaeological sample from industrial Coventry, what kind of potential pollution would have been the individuals exposed to that could justify the behaviour of AEFC in relation to the presence of smoking damage?

Other minor comments:

-Figure legend for figure 1 needs to say what context number/skeleton number it is

-Line 244: although technically you can start a sentence with a number ‘8/24’ it doesn’t look appropriate for a formal paper to do it.
